# Cerebrolysin and early neurorehabilitation in patients with acute ischemic stroke: a prospective, randomized, placebo-controlled clinical study

**Published:** 2017

**Authors:** A Stan, C Birle, A Blesneag, M Iancu

**Affiliations:** * Department of Clinical Neurosciences, “Iuliu Hatieganu” University of Medicine and Pharmacy, Cluj-Napoca, Romania; ** RoNeuro Institute for Neurological Research and Diagnostic, Cluj-Napoca, Romania; *** Department of Medical Informatics and Biostatistics, Iuliu Hatieganu” University of Medicine and Pharmacy, Cluj-Napoca, Romania

**Keywords:** early neurorehabilitation, neurorecovery, Cerebrolysin, acute stroke, neurotrophic factors

## Abstract

**Background** - Stroke represents one of the most important causes of permanent physical or mental disability. A number of recent advances in recovery have reinforced the idea that pharmacological intervention combined with a specific rehabilitation therapy can reduce disability after stroke.

**Objective** - The aim of this trial was to demonstrate the hypothesis that the association of pharmacological treatment with Cerebrolysin to early physical therapy can significantly stimulate the endogenous processes underlying the recovery after an ischemic stroke.

**Methods and Results** – It was a prospective, randomized, double-blind, placebo-controlled clinical study. 60 patients were randomized either to 30 ml/ day Cerebrolysin or to Placebo for 10 consecutive days, starting in the first 24-48 hours after stroke. The pharmacological treatment was paired with early physical rehabilitation. The robust nonparametric evaluation of the National Institute for Health Stroke Scale (NIHSS) demonstrated a large superiority of Cerebrolysin relative to placebo on day 10 with a MW=0.79 (95% CI, 0.65-0.94), respectively on day 30 with MW=0.75 (95% CI, 0.60-0.89). Similar results were found with modified Ranking Scale (mRS) and Barthel Index (BI). Cerebrolysin was safe and well tolerated.

**Conclusions** – Cerebrolysin had a beneficial effect on global neurological status and disability. The beneficial results of this study can be easily applied in the current clinical practice.

**Abbreviations:** BI = Barthel Index; CB = Changes from Baseline; CI = Confidence interval; ICH = International Conference on Harmonization; ITT = intention-to-treat; LB = Lower Bound of Confidence Interval; mRS = modified Rankin Scale; MW = Mann-Whitney; NIHSS = National Institute for Health Stroke Scale; P = P-value; R = Valid Number Reference Group (Placebo); SD = standard deviation; T = Valid Number Test Group (Cerebrolysin); UB = Upper Bound of Confidence Interval

## Introduction

Stroke represents one of the most important causes of permanent physical or mental disability [**1**]. In the Eastern Europe countries, the mortality caused by stroke is 6-7 times higher than in Western countries [**2**]. The dramatic impact of stroke on the individual, family and medical resources is accentuated by major debilitating complications and long-term repercussions on mental and physical functions [**1**][**2**]. A huge number of clinical trials focused on the treatment of vascular reperfusion and neuroprotective treatment in the acute phase, while early neurorehabilitation remained in the shadow. In the context in which only a limited number of patients benefit from revascularization treatment, the support of innovative research aimed to stimulate recovery after stroke, different from vascular reperfusion and applied in an extended time window being of major importance. A number of recent advances in recovery after stroke have reinforced the idea that pharmacological intervention combined with a specific rehabilitation therapy can reduce disability after stroke [**3**]. This was also the purpose of our clinical study, whose primary objective was to demonstrate the hypothesis that the association of pharmacological agents, such as Cerebrolysin, with early physical therapy, can significantly stimulate the endogenous processes underlying the recovery after a stroke. Neuroplasticity encompasses all mechanisms of neuronal reorganization, the most common being synaptogenesis, dendritic growth, axonal sprouting, recruitment of new anatomical pathways with similar functions to those injured, activation of functional but silent synapses and angiogenesis. The common promoter of all these processes is the presence of neurotrophic factors and vascular growth factors. The ability of the brain to reorganize itself after stroke through neuroplasticity can be modulated by pharmacological and nonpharmacological therapies [**4**][**5**][**6**][**7**][**8**][**9**][**10**]. 

Physical rehabilitation was identified as a key component of early neurorehabilitation. Although all clinical guidelines support early neurorehabilitation, its initiation time, duration and intensity have not yet been clearly defined. For most authors, this involves the initiation of recovery procedures within the first 30 days after stroke. The rehabilitation initiated between 30-180 days is defined as late rehabilitation, and the one initiated after 180 days is called chronic rehabilitation [**10**].

The most studied drugs in early rehabilitation after stroke selective serotonin reuptake inhibitors and Cerebrolysin. 

Chollet et al. published the results of the randomized, double-blind, placebo-controlled study "Fluoxetine on Motor Rehabilitation after Ischemic Stroke" (FLAME), in which 118 patients with acute ischemic stroke were included. Physical therapy was introduced in the first 5-10 days after stroke onset, and was associated with either 20 mg fluoxetine or with placebo. There was a significant improvement of motor deficit in the fluoxetine group at 90 days, measured using Fugl-Meyer motor scale scores (adjusted mean score=4.0 points; (95% confidence interval (CI) 29.7–38.4) compared to the placebo group (adjusted mean score=24.3 points, (95% CI 19.9-28.7); p=0.003) [**12**].

Acler et al. studied the effect of 10 mg Citalopram versus placebo, associated with physical therapy in early rehabilitation after stroke. After 4 weeks of treatment, the authors found an improved neurologic status, measured by the National Institute for Health Stroke Scale (NIHSS), in the Citalopram group, compared to the placebo group (p=0.03), as well as decreased excitability of the unaffected hemisphere, measured by transcranial magnetic stimulation, compared to the placebo group [**13**].

Recently, Muresanu et al. published the results of a prospective, randomized, double-blind, placebo-controlled, multicenter study, “Cerebrolysin and Recovery after Stroke (CARS)” [14]. The authors compared the effects of 30 ml Cerebrolysin versus placebo during early rehabilitation after stroke. The study drug was administered once daily for 21 days, beginning at 24 to 72 hours after onset. The patients also participated in a standardized physical rehabilitation program of 2 hours per day for 21 days. The patients from Cerebrolysin group had a better motor recovery measured using Action Research Arm Test score on day 90 (Mann–Whitney (MW) estimator, 0.71; 95% CI, 0.63–0.79; P<0.0001) [**14**]. 

Cerebrolysin is a multimodal, pharmacological agent with neurorestorative and neuroprotective effects, which mimics the action of neurotrophic factors. It stimulates neuronal survival and differentiation, axonal growth and sprouting, the formation of new synapses, and neurogenesis in the dentate gyrus [**15**][**16**]. Cerebrolysin is a peptide preparation produced by a biotechnological process, a standardized enzymatic breakdown of purified, lipid-free brain proteins. It consists of low molecular weight neuropeptides (<10kDa) and free amino acids. Cerebrolysin is produced by EVER Neuro Pharma GmbH, Austria and it is approved for the treatment of stroke, traumatic brain injuries and dementia in a number of European and Asian countries [**15**].

## Methods

**Study Design and Treatment Regimen**

We performed a prospective, randomized, double-blind, placebo-controlled clinical study evaluating the effects of Cerebrolysin versus placebo during early rehabilitation after stroke. First group (Cerebrolysin group) received 30 ml Cerebrolysin for 10 consecutive days and the second group (placebo group) received 30 ml placebo for 10 days. Both groups performed early rehabilitation. The treatment was introduced in the first 24-48 hours after stroke onset. Cerebrolysin was administered in a single daily dose diluted with 0.9% saline solution to a total volume of 250 ml. It was given as an intravenous infusion over a period of 60 minutes, at approximately the same hour every day. An identical amount of 0.9% saline solution (250 ml) was used as placebo.

For all patients, the rehabilitation program started immediately after the first study drug infusion for 10 consecutive days. It consisted of 2 hours per day of massage, passive and active movements for each patient. The number of repetitions and the complexity of the procedures were tailored to the level of performance of each patient, in order to avoid fatigue. Each patient performed four study visits. Screening and baseline visits were done in the first 24-48 hours after stroke, before the administration of the study drug. The next visit was conducted at the end of the treatment (10 days) and the last visit was done at 30 days after stroke onset.

The study was performed in the Neurology Clinique, Emergency County Hospital Cluj-Napoca, Romania. The relevant institutional ethics committees approved the study and all subjects signed an informed consent form. Patients who were not able to understand the informed consent were not included in the study. All the study procedures were conducted in accordance with the applicable laws and guidelines, Good Clinical Practice, and ethical standards.

**Inclusion and Exclusion Criteria**

**Inclusion criteria** were: (1) The onset of ischemic stroke with 24-48 hours before the first study drug administration; (2) Supratentorial ischemic stroke confirmed radiographically (brain magnetic resonance imaging) with a volume of over 2 cc; (3) No other stroke within the last 3 months; (4) Age between 18 and 80 years; (5) No significant pre stroke disability (modified Rankin Scale (mRS) 0 or 1 before the index stroke).

**Exclusion criteria** were: (1) Patients with complete remission of symptoms in the early hours after onset; (2) Patients with progressive stroke; (3) Patients with impaired consciousness (coma and stupor); (4) Patients with hemorrhagic or subtentorial stroke; (5) Patients with other severe comorbidities like uncontrolled hypertension, severe heart or lung diseases, moderate or severe dementia before stroke, malignancy, chronic liver disease, severe renal impairment, etc; (6) Patients with other neurological diseases that interfere with neurological assessment; (7) Pregnancy and lactation; (8) The patients included in other clinical trials.

**Randomization and Blinding**

Patients were randomly assigned in a 1:1 ratio to either Cerebrolysin or placebo using a computer random-number generator. An external body, independent of all study-specific procedures, created the randomization codes. Authorized healthcare providers responsible for the preparation of study medication to ensure concealed allocation used a sealed opaque treatment envelope system. These healthcare providers were independent of any other study specific procedures, particularly the efficacy and safety evaluations.

Neither the clinical examiners nor the patients knew which randomization group the patient belonged to.

**Efficacy criteria**

The primary efficacy criterion was the change in the global neurological status measured using NIHSS score from baseline to day 30. The secondary efficacy criteria were the changes in the activities of daily living, measured using mRS (1) and Barthel Index (BI) (2) from baseline to day 30.

**Statistical Method**

The analyses based on nonparametric approach were performed using the TESTIMATE v.6.5.14 software package on high security computers within a validated working environment. Standard descriptive summary statistics were calculated for the rating scales: arithmetic mean, standard deviation, minimum value, lower quartile, median, upper quartile, maximum value, number of non-missing values. Although this study was originally intended to be of exploratory nature, the first line analysis of this nonparametric approach is based on “confirmatory” principles. The first line nonparametric analysis was performed using the exact Wilcoxon-Mann-Whitney test. The multiple level alpha of the study (global level of significance for the whole study) was defined as alpha = 0.05, two-sided test for superiority.

According to the ICH Guideline E9 (ICH Topic E9, Statistical Principles for Clinical Trials, Step 4, Consensus guideline, 5 February 1998, CPMP/ICH/363/96), the results are given as P-values, as well as effect size measures with their confidence intervals (Mann-Whitney effect size as effect size measure related to the Wilcoxon-Mann-Whitney test [**17**]), so that the direction and quantity of the treatment effects are determined with their precision. Multiplicity was controlled by means of the principle of a priori ordered hypotheses [**18**] (1: NIHSS, 2: mRS, 3: BI). The analyses were performed with the intention-to-treat (ITT) population according to the ICH Guideline E9. The full analysis set (ITT set) was represented by all the randomized patients who received at least one dose of study medication with a primary endpoint assessment (NIHSS score) at baseline and at least one assessment after the first dose. All randomized patients who received at least one dose of study medication formed the safety analysis set.

## Results

**Study population**

A total of 60 patients with acute ischemic stroke were enrolled in this study (Cerebrolysin, n=30, placebo, n=30). All patients received at least one dose of study drug and they represent the safety analysis set. One patient from the placebo group prematurely discontinued the study because he left the hospital and voluntary withdrawn the consent. The ITT and per protocol analysis sets comprised 59 patients. Groups were well comparable with respect to baseline characteristics (**Table 1**) and prevalence of vascular risk factors (**Table 2**). There were no significant differences between the groups.

**Table 1:** Demographic baseline characteristics (safety analysis set) SD-standard deviation

Parameter		Cerebrolysin(N=30)	Placebo(N=30)	Total(N=60)
**Age (Years)**	Mean (SD)	62.96 (10.9)	65.24 (11.1)	64.30 (11.1)
**Sex**	Male (%)	19 (63.3)	20 (66.5)	39 (65)
	Female (%)	11 (36.7)	10 (33.3)	21 (35)
**Weight (kg)**	Mean	81.6	82.2	81.9
**Height (cm)**	Mean	169.2	170.3	169.75
**Dominant hand**	Right	29	30	59
	Left	1	0	1

**Table 2:** The prevalence of vascular risk factors in the groups (safety analysis set)

		Cerebrolysin (n=30)	Placebo (n=30)	Total (n=30)
Hypertension	n, %	21 (70)	18 (60)	39 (65)
Atrial Fibrillation	n, %	7 (23.3)	6 (20)	13 (21.6)
Dyslipidemia	n, %	18 (60)	16 (53.33)	34 (56.6)
Obesity	n, %	21 (70)	23 (76.6)	44 (73.3)
Past smokers	n, %	14 (46.6)	11 (36.6)	25 (41.6)
Current smokers	n, %	12 (40)	14 (46,6)	26 (43.3)
Diabetes mellitus	n, %	6 (20)	4 (13.3)	10 (16.6)

**Primary efficacy criterion (NIHSS score)**

No significant difference was also noted regarding baseline distribution of NIHSS scores (Mann-Whitney test, MW = 0.39, p=0.138), however, the NIHSS baseline scores were slightly higher in the Cerebrolysin group (Cerebrolysin group: 8.9±3.42 (mean±SD), placebo group: 7.8±2.36). The mean absolute decrease in the NIHSS score on day 30 was -6.1±2.27 in the Cerebrolysin group as compared to -4.0± 2.27 in the placebo group.

**Fig. 1:**
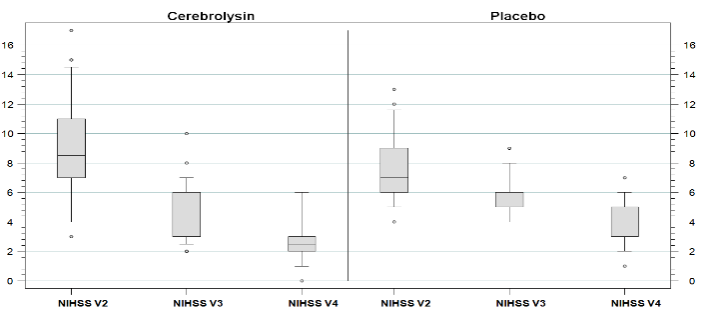
Evolution of NIHSS scores in both groups

The evolution of NIHSS scores in both groups is represented in **Fig. 1** (Boxplot, P10, P90).

**Fig. 2:**
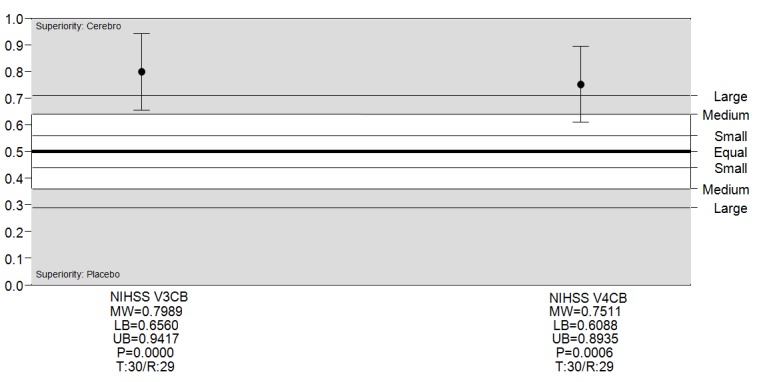
Effect sizes (Mann-Whitney, MW) of the NIHSS score changes from baseline in the ITT analysis

Abbreviations: Changes from Baseline (CB), Lower Bound of Confidence Interval (LB), Upper Bound of Confidence Interval (UB), P-value (P), Valid Number Test Group (Cerebrolysin) (T), Valid Number Reference Group (placebo) (R)

The robust nonparametric evaluation demonstrated a large superiority of Cerebrolysin relative to placebo on day 10 with a MW=0.79 (95% CI, 0.65-0.94), respectively on day 30 with MW=0.75 (95% CI, 0.60-0.89) (**Fig. 2**).

**Secondary efficacy criteria**

As in the case of NIHSS, no significant baseline differences were noted regarding the distribution of mRS scores (Mann-Whitney test, MW = 0.39, p=0.113), and BI scores (Mann-Whitney test, MW = 0.46, p=0.615) between the groups. The mRS and BI scores in different time points of the study, together with their changes are presented in the **Table 3** and **4**.

**Table 3:** mRS scores evolution and changes

mRS	Cerebrolysin, n=30, mean ± SD	Placebo, n=29, mean ± SD
Baseline (V2)	4.2 ± 0.82	3.9 ± 0.7
Day 10 (V3)	2.7 ± 0.95	3.3 ± 0.65
Day 30 (V4)	1.9 ± 0.9	2.4 ± 0.94
Changes in mRS score comparing with baseline		
Day 30	-2.4 ± 0.76	-1.6 ± 0.91
Day 10	-1.5 ± 0.9	-0.7 ± 0.55

**Table 4:** IB scores evolution and changes

BI	Cerebrolysin, n=30, mean ± SD	Placebo, n=29 mean ± SD
Baseline (V2)	27.0 ± 27.09	30.3 ± 25.56
Day 10 (V3)	65.2 ± 20.49	53.8 ± 19.76
Day 30 (V4)	85.7 ± 14.06	73.6 ± 18.80
Changes in IB score comparing with baseline		
Day 30	58.7 ± 22.09	43.3 ± 21.48
Day 10	38.2 ± 18.73	23.4 ± 14.77

Analysis of the distribution of the mRS score at 30 days showed that the percentage of independent patients at 30 days (mRS = 0, 1, or 2) was 73.33% in the Cerebrolysin group as compared to 44.83% in the placebo group.

**Fig. 3:**
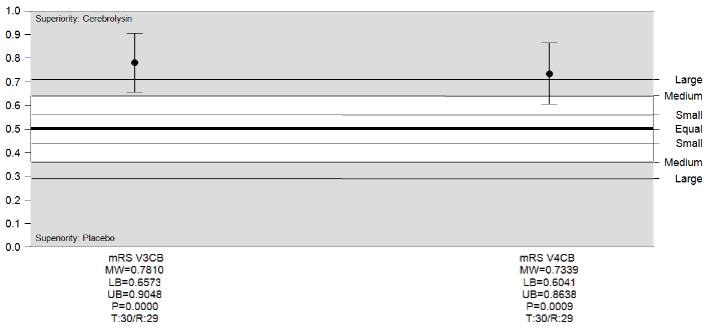
mRS score changes from baseline, Cerebrolysin vs. Placebo, ITT. Analyses were conducted using Wilcoxon-Mann-Whitney Test

The nonparametric evaluation of mRS scores demonstrated a large superiority of Cerebrolysin relative to placebo on day 10 with a MW=0.78 (95% CI, 0.65-0.90), respectively on day 30 with MW=0.73 (95% CI, 0.60-0.86) (**Fig. 3**). Despite the slightly worse baseline severity in the Cerebrolysin group, also the final absolute values well demonstrated the superiority of Cerebrolysin with MW=0.65 (95% CI, 0.51-0.78).

**Fig. 4:**
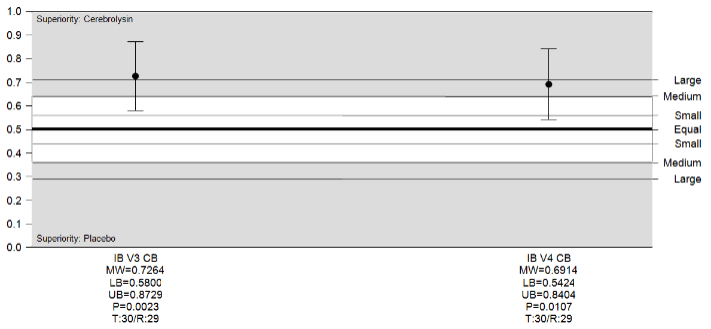
BI changes from baseline, Cerebrolysin vs. Placebo, ITT. Analyses were conducted using Wilcoxon-Mann-Whitney Test

Regarding BI, a medium superiority of Cerebrolysin versus placebo was found using Wilcoxon-Mann-Whitney test on day 10 with a MW=0.72 (95% CI, 0.58-0.87), respectively on day 30 with MW=0.69 (95% CI, 0.54-0.84) (**Fig. 4**).

There were no significant differences in the rate of acute complications between the groups. The majority of adverse events (79.37%) were rated as mild in severity. One patient from the placebo group experienced a serious adverse event described as bronchopneumonia resolved during the study period. No patient died. The adverse events are listed in **Table 5**.

**Table 5:** The frequency of adverse events in both groups, safety analysis set

Adverse events, n (%)		Cerebrolysin (n=30)	Placebo (n=30)
Bronchopneumonia	n, %	1 (3.33)	2 (6.66)
Deep venous thrombosis	n, %	1 (3.33)	0 (0)
Urinary infections	n, %	7 (23.33)	7 (23.33)
Pressure ulcers	n, %	0 (0)	1 (3.33)
Falls	n, %	3 (10)	2 (6.66)
Depression	n, %	2 (6.66)	3 (10)
Psychomotor agitation	n, %	2 (6.66)	1 (3.33)
Sleep disturbances	n, %	4 (13.33)	4 (13.33)
Hypertension	n, %	6 (20)	5 (16.66)
Stable angina	n, %	1 (3.33)	1 (3.33)
Abdominal pains	n, %	3 (10)	4 (13.33)
Catheter phlebitis	n, %	7 (23.33)	8 (26.66)
Articular pains	n, %	3 (10)	4 (13.33)
Total		40	42

## Discussions

The combination of Cerebrolysin with early physical neurorehabilitation contributed to a significant improvement of global neurological status, measured using NIHSS, both at 30 (p=0.0127) and 10 days (p=0.0075), compared to the early rehabilitation alone. These results confirmed the primary objective of our study. We included patients with moderate stroke severity (mean NIHSS=8.9). At 30 days, there was a 6.1 points improvement of the mean NIHSS score in the Cerebrolysin group, and only 4.0 points in the placebo group. These good results of patients from the Cerebrolysin group were also seen in the CASTA study at 3 months after onset [**20**] suggesting that the association between Cerebrolysin and early rehabilitation can speed up the recovery processes. Our results are comparable with those from previous early rehab trials with Cerebrolysin [**14**][**19**].

A significant improvement in disability at 30 days, measured with mRS, was noted in the Cerebrolysin group (p^lt;0.001), compared to the placebo group. The high percentage of patients having a mRS score of 0, 1 or 2 at 30 days in the Cerebrolysin group (73.3%) compared to the placebo group (44.83%) showed that more patients from the Cerebrolysin group regained their walking independency and were able to carry out all the usual duties and activities. According to literature, 60-80% of the stroke survivors are able to walk without support after 6 months [**21**][**22**]. In our study, after just 30 days, 73.33% of the patients in the Cerebrolysin group were fully ambulatory, suggesting that Cerebrolysin significantly enhances the neurorecovery after stroke. Our results are similar to those found in the CARS trial [**19**].

BI scores predominantly reflect the improvements in lower limb deficit. At 30 days, a significant superiority was noted in the Cerebrolysin group compared to placebo, results that reinforce the data found on the previous two clinical scales.

A special attention should be paid to concomitant medical conditions and to modifiable environmental factors that may interfere with the recovery process. Medical history such as cardiovascular and pulmonary diseases, advanced rheumatic diseases, fever, depression, sleep disorders, incontinence, or acute urinary retention may significantly reduce the functional prognosis of the patient with stroke [**21**]. In our study, there were no significant differences between the groups in the comorbidity rate, and the rate of acute complications following stroke was similar in the Cerebrolysin and placebo group.

The rate of complications in our study was lower than that reported in other clinical trials, perhaps due to the shorter surveillance period of the patients [**14**][**23**][**24**][**25**]. Also, early rehabilitation reduces the risk of acute complications after stroke. Association of Cerebrolysin to early neurorehabilitation is a safe method for patients.

The limitations of our study are related to the small number of patients, ischemic strokes type heterogeneity, and short duration of the study. Even so, it is known that NIHSS is a good predictor for long-term recovery so we can anticipate the good prognosis of patients from the Cerebrolysin group [**26**]. The strength of our study is that it was a prospective, randomized clinical trial with robust and of high clinical relevance results.

## Conclusions

Our study is a small-randomized clinical trial that had only an exploratory role in order to create valid hypotheses that could underlie future researches. Bigger clinical trials are needed to find answers to the many remaining questions related to the therapeutic doses and the critical time window where neuroplasticity can be stimulated, and in particular related to the need of personalized recovery therapy. The beneficial results of this study can be easily applied in the current clinical practice as the drug is approved for stroke treatment, it is well tolerated, requires no sophisticated administration procedures, and the early physical neurorehabilitation can be performed without significant extra costs.

## Conflict of interest

The authors declare no conflict of interest.
